# Mass digitization of scientific collections: New opportunities to transform the use of biological specimens and underwrite biodiversity science

**DOI:** 10.3897/zookeys.209.3313

**Published:** 2012-07-20

**Authors:** Reed S. Beaman, Nico Cellinese

**Affiliations:** 1 Florida Museum of Natural History, University of Florida, Dickinson Hall, Museum Rd, Gainesville, Florida 32611-7800, U.S.A.

**Keywords:** Scientific collections, biodiversity, digitization, specimen access, biodiversity informatics, data sharing, linked data, interoperability

## Abstract

New information technologies have enabled the scientific collections community and its stakeholders to adapt, adopt, and leverage novel approaches for a nearly 300 years old scientific discipline. Now, few can credibly question the transformational impact of technology on efforts to digitize scientific collections, as IT now reaches into almost every nook and cranny of society. Five to ten years ago this was not the case. Digitization is an activity that museums and academic institutions increasingly recognize, though many still do not embrace, as a means to boost the impact of collections to research and society through improved access. The acquisition and use of scientific collections is a global endeavor, and digitization enhances their value by improved access to core biodiversity information, increases use, relevance and potential downstream value, for example, in the management of natural resources, policy development, food security, and planetary and human health. This paper examines new opportunities to design and implement infrastructure that will support not just mass digitization efforts, but also a broad range of research on biological diversity and physical sciences in order to make scientific collections increasingly relevant to societal needs and interest.

## Introduction

Understanding biodiversity is one of five grand challenges identified by US National Research Council Committee on Forefronts of Science at the Interface of Physical and Life Sciences (2010). Broadly defined, the study of biodiversity addresses variation among living things and systems, ranging in scale from molecules, genes, cells, individual organisms, to species through ecosystems. Specimens, and now the digital proxies for specimens, are a critical underpinning in documenting biodiversity (Berendsohn and Seltmann 2010, Berents et al. 2010, Scoble 2010, Vollmar et al. 2010). Improving infrastructure for digital specimen data comes at a time when basic biodiversity science is itself undergoing rapid change.

Investments in digitization will ultimately yield a better return if use expands and specimen data are linked across a wide array of related biotic and abiotic data. The specimen objects provide a physical basis for linking data to other biodiversity science domains. Scientific collections document the who, what, where, and when of biological diversity. Digitization, beyond making collections more accessible to researchers, provides access to downstream users such as the general public, government and non-government agencies and private enterprises.

Many researchers still fail to realize the importance of vouchering specimens to their community’s practice. Whether they study molecules or ecosystems, many are content to document the organisms they work with by taxonomic name alone. Even researchers in the closely aligned field of molecular systematics have previously failed to grasp the importance of citing specimen vouchers, evidenced, for example, in the lack of voucher data cited in GenBank, other repositories, and in publications. How can we know that the sequence deposited in GenBank belongs to the taxon under which it is filed? Whether alpha taxonomy or a synthesis of large phylogenetic trees based on molecular sequences, citing vouchers remains essential to a scientific process that is repeatable and verifiable.

In order for research communities to stay abreast and benefit from opportunities of new information technology environments (e.g., cloud computing, linked data and ontologies, social and computational virtual networks), increasing multi-disciplinary collaboration between biologists and computer and information scientists and engineers is a must, as few scientists in representative domains have all the necessary skills to “do it all.” Across the biological sciences, where new tools such as next generation sequencing and environmental sensors challenge network design and contribute to the now well-known data deluge (Kahn 2011, McNally et al. 2012, Michener and Jones 2012, Kolker et al. 2012), robust cyberinfrastructure that facilitates collaboration, data automation, sustainable software development, and high performance computing is a priority (Donoghue et al. 2009, Hendry 2010). Digitization of scientific collections is no exception, as two- and three-dimensional images, video, audio, and other media derived from physical specimens and observations and measurements proliferate, they add significantly to the data deluge, and to the need for long-term data storage archives and data curation. It is also essential to recognize that digitized collections permanently document resources that are held in museums and herbaria, and so have a place in foundational biodiversity infrastructure.

Some of the necessary organizations are already in place, e.g., Global Biodiversity Information facility (GBIF: http://www.gbif.org ), Atlas of Living Australia (ALA: http://www.ala.org.au ), Virtual Biodiversity Research and Access Network for Taxonomy (ViBRANT: http://vbrant.eu ), DataONE (http://dataone.org ), and the US Integrated Digitized Biocollections (iDigBio: https://www.idigbio.org ), which are at various stages of implementation and operation. Each, however, has limitations on scope, and the resulting infrastructure remains an innovative yet incomplete patchwork of distributed data, archival resources, tools and software. For example, GBIF has no mandate as a primary resource provider, and instead serves as an aggregator, indexer, and distributed portal; iDigBio is not funded to develop new digitization tools, and like ALA has a national mandate.

The gaps in scope present both a need and opportunity to further conceptualize and develop an international infrastructure and missing components that will fully support the broad definition of biodiversity research that coordinates and integrates with existing infrastructure, including tools developed by individuals and small teams. Coordinating biodiversity research and cyberinfrastructure requires nimble computational resources, an ability to support heterogeneous distributed data, robust and sustainable software development, and an innovative and well-trained workforce, along with the social and research infrastructure that supports them, to answer challenges that have previously been beyond the scope of traditional scientific methods and organizations.

This paper is a call to the community to define a comprehensive conceptual plan that will allow scientists across multiple disciplines to coordinate a community able to capitalize on cutting edge computational infrastructure, economies of scale, with the innovation and needs of a broad community of other scientific organizations. So far, the biocollections community has operated in an ad hoc, geographically fragmented way. As research has become increasingly collaborative, interdisciplinary, and international, new social challenges arise around how scientists work together, across disciplines, institutions, and geographic and political boundaries. Community based planning allows consideration of critical elements of sustainable infrastructure, including:

• Setting priorities and identifying use cases.

• Identifying stakeholders, collaborators, and communities of practice.

• Specifying computational infrastructure, software, and data storage requirements and dependencies.

• Practices, methods, standards, and interoperability.

• Management, organizational structure, and sustainability.

• Risk assessment.

Formal conceptual planning and development of standards is common in engineering, industrial, and biomedical sectors, but in basic biological research, a perception remains that innovation and individual research are not as dependent on foundational infrastructure as in the physical sciences. As networks of biodiversity researchers grow, they have an increased need to plan effective infrastructure to support collaboration, distributed data management and access. As an example, extensive planning and design processes are documented in a NASA (2007) handbook on systems engineering, including lifecycle documentation, establishing user requirements, and management. The elements listed above and discussed below are not exhaustive, and are described in a context of how digitized collections can underwrite a larger community in the biodiversity sciences.

## Priorities and use cases

A challenge of scale for this community is in the numbers. Over a billion specimens exist in thousands of collections, and most are managed independently within stand-alone museums, universities, and government agencies (http://nscalliance.org/wordpress/wp-content/uploads/2009/11/iwgsc-report.pdf ). Digitizing an institution’s collection from A-Z may be the most efficient means, but feasible only in certain circumstances, such as large-scale moves or renovations (e.g., the recent renovation of the Paris Herbarium). Funds, personnel, and time are typically limiting, so priorities must be set. Type collections, historical collections, special collections are common priorities, but identifying and increasing relevance of collections to the research community and other stakeholders is another strategy.

The aggregation of digital data through portal infrastructure such as the Global Biodiversity Information Facility (GBIF: http://www.gbif.org ), VertNet (http://vertnet.org ), Morphbank (http://www.morphbank.net ), the Paleontology Portal (Paleoportal: http://www.paleoportal.org ), among others, added to the realization that specimens are useful for much more than simple mapping of species occurrences. Digital specimen data is a proxy or surrogate of physical objects and appropriate use may be limited. However, digitized data can be used to study morphology (Corney et al. 2012), identify, classify, map and spatially model taxa (Thuiller et al. 2009, Soberón 2010). Where expertise is a limiting resource, for example in the study of hyper diverse groups (e.g., insects), cyberinfrastructure can help leverage that expertise (Moore 2011).

There is further need to establish specific use cases (or more precisely, user scenarios) whether biological, technical, or a combination of both. As applied to collections digitization or other areas of biological informatics (e.g., genomics and proteomics), research is increasingly catalyzed by improved computational infrastructure to process and store large data sets and files, index and link billions of data records, data-mine existing resources, and incorporate ontologies to support semantic reasoning. Engineering breakthroughs in optical sensors and robotics have had and will continue to have enormous potential to guide and impact digitization efforts, but the needs of the biology domain can also drive technology.

## Stakeholders, collaborators, and communities of practice

Stakeholders, both primary users (e.g., curators, collection managers) and downstream users (e.g., climate researchers, resource managers, educators), are the most appropriate source of user scenarios. It is the stakeholders that build communities of practice from the ground up and define what is really needed, what is novel, and add value to current practice. Users define the need to scale infrastructure capabilities to support the science (e.g., geospatial and phylogenetic analyses). Users also compose the social networks, crowd-sourcing workforce, and ultimately provide intellectual capacity for digital markup and annotations, development of linked data applications, ontologies, automation, and workflows.

In 2010, the scientific collections community within the United States outlined a strategic plan for digitizing scientific collections, including the establishment of the Network Integrated Biocollections Alliance (NIBA, http://digbiocol.wordpress.com ). The plan defined digitization to encompass a broad range of digital data capture about biological specimens, from field collection events to cataloging and accessioning metadata, images and other media derived from field and laboratory work, and set the stage for establishing priorities based upon how a specimen and its occurrence relate to research. Additionally, the physical specimens can be re-sampled, e.g., for epiphytes, parasites, mineral deposits, bio-medically active compounds, re-purposing not just data, but the specimen objects themselves, for research on many functional elements of biodiversity, including mutualism, co-evolution, lateral gene transfer, parasitology, and community ecology.

The U.S. National Science Foundation responded to elements of the NIBA plan by establishing a program for Advancing Digitization of Biological Collections (NSF-ADBC, http://www.nsf.gov/funding/pgm_summ.jsp?pims_id=503559 ), which funds digitization based on scientific questions or themes through extensive collaborative networks. Examples of Thematic Collection Networks (TCN) funded through this program are detailed on the iDigBio web site (https://www.idigbio.org/content/thematic-collections-networks ).

Key challenges are often social and priorities may be at odds with technical needs. Solving social challenges requires different approaches and expertise not be inherently a part of existing biocollections business practices. Long adhered to curation practices may need to be revised, and interdisciplinary collaboration with social scientists and psychologists may provide useful insight, but may not necessarily be well received. For example, is it legitimate to unpin an insect to access the label data during the digitization process? As investments in digitization increase, so will the need to produce metrics of success and document outcomes. As communities of practice develop around digitization networks, social and usability considerations are essential.

## Computational infrastructure

Computing, software, and data resources are clear enablers of both large-scale digitization and biodiversity research. Advance computational infrastructure, including virtual and cloud infrastructure, are costly to design and deploy, so are generally viewed as resources to be adopted across all sciences. In the U.S., the nationally funded TeraGrid, and its successor XSEDE, have primarily focused on processing capability, or cycles, and benefits applications such as phylogenetic inference, image manipulation, analysis and visualization, but less so for the storage requirements of digital collections, including long-term archiving of images and other media.

Dependencies often relate to previous investments and software development in the form of libraries, services, and value added data sets. Georeferencing tools, e.g., GeoLocate (http://www.museum.tulane.edu/geolocate ), are good examples of existing investment that incorporates automation, data-mining algorithms, need for gazetteer and other geospatial data, and mapping tools. Automated data capture methods, for example the use of Optical Character Recognition (OCR) may leverage commercial software and allow deployment of services or software with embedded OCR.

## Practices, methods, and workflows

Digitization workflows span across human mediated processes through data and computationally intensive automation where software tools and services are the actors and intersect field collection techniques, institutional accession policy, differences in curatorial practice among domains, and involvement of the general public in crowd-sourced methods.

The workflows that represent digitization of new accessions have in many cases required, or at least highly recommended, elements of funded projects in systematics and ecology. The Moorea Biocode project (http://moorea.berkeley.edu/biocode ) is an exemplar, comprehensive effort to collect data on all aspects of a biodiversity survey, including vouchers, tissues, photos and other media. Expanding on efforts such as this has potential to test capacity for digitization and physical curation. BioBlitzes are similar approaches that typically utilize a combination of expert and citizen scientists over a short period of time (a day or few).

Digitization of existing collections is an enormous undertaking. Initial digitization efforts focused on assembling very complete data records and access to researchers and the public was granted only after extensive quality control. More recently, it has been recognized that not every element of a collection record needs to be recorded in a single digitization event (Granzow-de la Cerda and Beach 2010). For example, recording of an image and “filed-under” taxon name are sufficient to start the process. Digital capture of useful information can follow at a later stage and be treated as annotations (e.g., a history of taxonomic determinations). Some aspects of data capture, like data curation, can be costly when it involves expert judgments. In entomology, for example, the initial capture of a box of specimens that may contain hundreds of individuals represents a further extension of a modular workflow. This works effectively with high-resolution sensors that allow users to scale their view appropriately.

Imaging methods have great growth potential for mass digitization efforts. Those new to digital imaging may find the array of possibilities overwhelming. Sensor resolution, pixel size, noise sensitivity, and cost are among the factors that must be weighed. Considering fitness for use means that there are no one-size-fits-all solutions; collections inherently vary in the ways that physical objects and their associated data are stored, and differ in size (from a few thousand to millions), use cases, and available budgets.

Another consideration that may ultimate affect use of a digital media objects are the formats in which they are stored, archived, and made available to researchers. Metadata, annotations, color profiles, etc. can be stored within the image, as in the case with EXIF metadata (Romero et al. 2008) or in separate databases. These presence and access to such metadata affect whether viewers can display certain media types, decode metadata, and access or provide new digital annotations. Whether the image formats are proprietary or open source, the type and level of file compression, e.g., lossless vs. lossy, are particularly important in biodiversity research applications, and especially when data are to be archived over the long-term.

## Standards and interoperability

Data sharing requires that the resources be communicated in standard formats, consistent usage of vocabulary and concepts, and through protocols understood by each of the nodes of a network. In the biodiversity domain, Darwin Core (DwC, http://rs.tdwg.org/dwc ), a TDWG supported standard (Wieczorek et al. 2012), is widely adopted, including by GBIF and it is used by many of GBIF’s data providers in the context of the Integrated Publishing Toolkit (IPT), a recently developed tool for easy data sharing (http://code.google.com/p/gbif-providertoolkit/ ). In its current instance, DwC is for all intents and purposes a controlled vocabulary of terms that describe scientific collections, biodiversity observations, basic taxonomies, and localities, among others. Concepts are defined in human readable language and implementations are independent from any one format (e.g., XML, RDF, or tab-delimited). This creates flexibility to link data from the collections to virtually any other digital record in related domains. Recent harmonization efforts, for example through the Genomic Standards Consortium (http://gensc.org/gc_wiki/index.php/Main_Page ), which is developing profiles for minimum information standards (MIxS), make it possible to link genomics data to scientific collections. While very preliminary, such efforts herald recognition that information needs to be exchanged across multiple domains in biology, geo-sciences, and other physical sciences.

Linked data environments are evolving quickly and increasing capacity for data discovery. A collection event may generate a number of specimens that are independently imaged and annotated; tissues may be subsampled from any specimen, its DNA extracted and sequenced. Specimens, annotations, images, tissue samples, DNA may be accessioned into collections at different institutions, and sequences deposited in GenBank. It is a challenge to track the data across different institutions, and especially across digital repositories in different domains. Linked data approaches can provide sufficient provenance to allow discovery of not just how a specimen may have been used, but if a digital annotation occurs (such as a change in identification) this can be propagated into downstream analyses. Projects like the BiSciCol Biological Science Collections Tracker (http://biscicol.blogspot.com ) aim at filling the gap in reconciling specimen data with their derivatives when these are scattered across independent digital repositories to support projects like Moorea Biocode. However, linked data approaches are successful only when data are served to the community and tracking can be achieved with the use of persistent Globally Unique Identifiers (GUIDs). As linked data efforts increase, it is becoming progressively evident that the persistence of GUIDs is both a necessity and a challenge. The responsibility of establishing a persistent GUID lies with the provider (see https://www.idigbio.org/content/idigbio-guid-statement ), although other scenarios that may include large data aggregators taking on the responsibility of assigning unique identifiers are also possible. In addition, identifiers need to be associated with individual data objects, and not just data sets.

The development of formal ontologies compliments and extends efforts on controlled vocabularies and linked data. Data modeling associated with ontologies can provide a powerful approach to synthesis in semantic web environments. The biomedical community has invested heavily in initiatives such as the Open Biological and Biomedical Ontologies (OBO Foundry, http://www.obofoundry.org ) and Gene Ontology (http://www.geneontology.org ). One advantage inherent to biocollections data is that a long history of practice has already led to structural understanding of ontological relationships, and biological classification has served as an example in the general literature on ontologies (Heuer and Hennig 2008). While relationships between collecting events, observations, organism occurrence, and taxonomy may never be solved in a philosophical context, in a pragmatic context, the definition of terms and the use of concepts may be more precisely aligned in shared data environments by consideration of ontological relationships. As the implementation of standards and the underlying terms and concepts is a matter of practice, technology may provide partial solutions, such as in the support of mapping semantic meaning across multiple ontologies and linked data environments.

## Risk assessment

While the promise of access and relevance to biological collections data are over-arching goals, digitization can also mitigate, to a very limited extent, the loss of physical collections. However, new field collections can never replace the original, especially when it comes to type specimens and historical collections, even if the localities from which they were collected still exist. Specimen acquisition, curation and preservation of specimens are an enormous long-term capital investment, and the digital capture and dissemination of data is a relatively minor cost in comparison.

Technology develops at such a rapid rate that long-term planning carries uncertainty and risk. For example, as digitization efforts begin to use cloud computing resources for data storage, they may not consider an element of vendor lock-in, i.e., that bandwidth costs may preclude them from migrating their data elsewhere. A related question is whether biodiversity data managers should even manage their own hardware resources, which often carry hidden costs such as system administration, electric power bills, and other needs that are often not scalable. Hardware lifespan is generally in the 3–5 year range, but carefully planned software and database designs can have much longer shelf life. Optimal methods to develop, maintain, and sustain software applications and data resources are not always clear, and even innovative tools focused on highly specific tasks (e.g., in genomics, proteomics, metabolomics) are unlikely to have a sufficient user base to gain commercial viability. In limited communities of practice, therefore, other business models such as subscription services are more likely to be sustainable in such cases. Collections are generally housed in organizations (museums and academic institutions) that already have a long-term commitment to their physical collections and are managed with public, private or endowed funding. Therefore, extending that commitment to digital information follows logically, but it should not be an unfunded mandate.

The potential for failure lurks around every corner. Many risks are social as much as technical. The individuals in the biodiversity research community may not be able to communicate user scenarios that are adequately understood by technical implementers. Additionally, potential collaborators may have conflicting needs, or may not have a sufficiently innovative vision to create opportunities in a multi-disciplinary environment. There are also significant challenges to broad adoption of digitized collections data, because users outside the immediate circle of formally trained scientists may not be interested in subtleties that drive extensive discussions in the biocollections community, e.g., taxonomic concepts. Downstream users, for example, often want to know only the names of the organisms they are sampling or studying.

## Conclusions

In recent years we have witnessed a renewed interest in natural history collections and with that, the leading edge of a deluge of digital biocollections data. Mass digitization approaches, driven by specific research questions, require a variety of methods tailored to the different nature of the specimens in question and requirements of the user scenarios. Rapid advances in technology allow us to implement a variety of tools and workflows that are well adapted to the needs of each collection, including specimen objects, methods of storage, available informatics and human resources. Mass digitization, no matter how achieved, offers the incredible opportunity for using biocollections to address and meet scientific grand challenges at small and large scale, within and across domains. The combination of human pressure on natural systems and new technologies for digitization creates a perfect storm of social imperatives and scientific opportunities to mobilize data and further explore under-described biodiversity still locked within museum cabinets.

The ultimate payoff for broad adoption of biocollection data resides in the synthesis of biodiversity data across domains spanning systematics, evolution, genetics, ecology, and to the physical and social sciences. If we link that knowledge only to a taxonomic name and not to a specimen, we are linking to a subjective judgment about an organism’s identity and not to the physical documentation of the organism itself. By linking experimental data to voucher specimens, experiments become more objective, repeatable, and the data gathered re-usable. Without the evidentiary documentation the investments in experimental research lose their value.

The massive amounts of digital data that we now generate are hard to manage or synthesize with lack of an appropriate infrastructure that helps tracking data provenance, metadata, and all specimen derivatives. This requires a cyberinfrastructure capable of accommodating multi institutional needs and a well-developed knowledge environment in which data can be easily synthesized and semantic reasoning applied. Two important messages arise, one social the other technical. First, in a broad, heterogeneous biodiversity research environment, we need a singular community effort to conceptualize and communicate necessary infrastructure at a larger scale than so far considered perhaps building upon the Global Biodiversity Informatics Conference (GBIC: http://links.gbif.org/supporting_biodiversity_science.pdf ) initiative via GBIF. Second, approaches in heterogeneous and distributed data environments that characterize biology require at a minimum persistent GUIDs associated with every specimen and digital data object. Metadata about collective data sets is insufficient. The digitization process is only part of a large data mobilization effort for biodiversity science. It is the very first step forward in order to make data discoverable and facilitate its synthesis.
